# Singular Case of Vicarious Menstruation

**Published:** 1811-01

**Authors:** John Kenworthy

**Affiliations:** Dobcross, Yorkshire


*To the Editors of the Medical and Physical Journal.*

Gentlemen,

On the 15th of March, 1809, a lady, 63 years of age, was attacked with pleuritis, but being from home when her friends sent for me, and detained by unavoidable circumstances until noon the day following, I consequently could not see her until the 16th about three o'clock in the afternoon. I found her in in a very distressed situation: the pain (which was on her left side) was violent; lancinating from the sternum to the scapula with great severity; inspiration remarkably difficult, increasing the pain so much as to induce her to scream out; pulse very hard and contracted, beating irregularly from 90 to 100 strokes in the minute; tongue covered with a thick white fur, except the sides of it, which were preternaturally red; bowels costive; urine scanty; thirst very distressing, but the pain occasioned by the effort of deglutition prevented her from alleviating it as she wished. I immediately took a pound of blood from her arm in as sudden a manner as possible, and induced syncope. The blood shewed no buff; the time of its flowing was about four minutes. I then directed the application of hot flannels to her side, until the arrival of a servant whom I had dispatched for a blister, and some powders composed of super tartras potassæ; nitratis potass. and gr. ¼. pulv. digitalis every third hour; immediately after the bleeding, she felt herself much relieved and breathed more freely; her pulse was much softer and fuller, and she now took copiously cooling acidulated drinks.

17. Symptoms varied very little since yesterday; urine high coloured; the blister rose well.

18. Pain much more violent; inspiration extremely difficult; pulse nearly in its original state, hard, tremulous, and contracted; cannot bear any other position than lying on her back with her head raised. Repeated the venæsection to a pound in the same sudden manner as before, and she was relieved; the blood was now covered with a thick opaque crust, and the surface was very concave; prescribed tart. antim. gr. ⅛ and ætheris nitros. gutt. xv. in mist. camphorat. with each dose of the powders as before.

19. 9 A. M. Has had no sleep, pain still acute, yet something more like a weight or fullness (as she expressed herself); bowels gently open, urine high coloured and scanty; pulse 80, a gentle moisture upon the skin; thirst very troublesome. Ordered six leeches to the side. Evening very restless, pain as in the morning, pulse 75, beating more regular and soft; prescribed a draft as follows, and left her for the night. ℞. Ext. hyosciam gr. x. ætheris nitros. oxymel colchici āā ʒj mist. camphorat. ℥j. M. H. S. S.

Monday morning, 20th. Had no rest, pulse compressed, 70—feels a great weight and pain in her left side quite up to her shoulder; if raised so as to sit up, she immediately becomes faint, and is obliged to recline on her pillows again; countenance expressing great anxiety, pale and dejected, except that on each cheek there is a circumscribed redness inclining to a purple hue; has not voided more than three ounces of urine since last night eight o'clock; suspecting an effusion within the chest, I prescribed as follows.

R. gum. gambogæ. gr. iv. ætheris nitros. oxym: colchici, tinct. scillæ. āā ʒiij. tinct. digitalis ʒij. mist. camph. aquæ puræ, āā ℥iiss. M sumat. cochl. dua 2a. qq. hora.

Repetatur H. anod. c. extra hyosciam. gr. xij. H. S. S.

21. Last night slept two or three hours, pain still severe, pulse 60 and compressed, intermitting at times one or two strokes; hands benumbed and cold, particularly on the left side; last night awoke from a dream much frightened, and was a long time before she could be composed; if she sits, faintness still immediately supervenes; has not voided more than six ounces of urine the preceding twenty-four hours.

Continue the mixture and draught at bed time as before.

22. Has had several fluid stools, and made about half a pint of water; fell asleep last night about twenty minutes after taking the draught, and remained so nearly two hours, when she awoke again in a fright and fancied she was suffocating; pulse 55, much compressed and intermitting frequently; hands and face œdamatous; the symptoms now evidently and distinctly mark the case to be hydrothorax.

Continue the mixture and draught as before.

23d and 24th. Much as before, except that the flow of urine is more copious and she has two fluid evacuations e recto in the twenty-four hours. Continue as before.

25th and 26th. Has voided about fourteen ounces of urine and two stools in the twenty-four hours, these last two days, which is somewhat less in quantity than what she takes; pulse 53, frequently intermitting; cannot bear an erect posture.

27th. A considerable remission of pain, pulse 55, compressed and low; she has made about a pint and a half of water the last day, and there is a free action of the bowels, so that the fluid discharged is nearly equal to the quantity drank. Continue as before.

30th. Flow of urine as before, a pint and a half; hands and face still œdematous, breathing very difficult, and she still awakes at times greatly alarmed and terrified; I added to her mixture, ætheris nitros. oxymel. colchici. tinct. scillæ āā ʒj. tinct. meloë vesicat. ʒij. to be taken as before with the draft at bed time.

April 5th. Urine discharged copiously; pulse 60, fuller and more regular, swelling of the hands and face subsiding; her breathing is not so confined, and she has slept tolerably composed the last two nights. What is to me most remarkable in this case is, that since the time of her last parturition, twenty-one years ago, she has never menstruated from the vagina, but invariably and regularly from the rectum, with this difference, that sometimes it has been to the amazing quantity of four, five, or even six pounds; yet this enormous discharge has not reduced her so low as might be expected, but merely kept her in a state of common debility. Notwithstanding her advanced age, the discharge still appears at regular periods, and is preceded by the common symptoms; a sudden check which was incautiously given to it, may, I think, be deemed the proximate cause of the pleuritis, in which form the system was first attacked; and consequently the serous effusion in the chest was the effect of the increased inflammatory action of the minute secreting arteries on the surface of the pleura; and with respect to the vicarious hæmorrhage, I cannot see any other method of accounting for it, than by supposing the principal branches of the uterine arteries obliterated, and some anastamosing branch of the hypogastric assuming the action, perhaps the hæmorrhoidea media.

April 11th. Hæmorrhage returned from the rectum; pulse 66, breathing much easier, can sit up four or five hours in the day; prescribed a mixture with extr. cinchon. solut. arsenic: and ætheris nitros. and to make free use of wine.

12th. Has had a restless night, hæmorrhage considerable, pulse 66; last night awoke from a dream frightened, swelling of the hands trifling, the quantity of urine voided is nearly double to the fluid drank, bowels freely open. Continue the cinchona, &c.

14th. A considerable pain in her head, pulse 70, natural, has a large flow of urine and one fluid evacuation e recto, per diem; nausea with an inclination to throw up; I consider it as the effect of the cinchona. She therefore omits it.

16th. Hæmorrhage stopped, pulse 70, natural, no thirst or pain, no sickness, appetite still poor; bowels regularly evacuated every day once, flow of urine copious; no swelling of the face or upper extremities. She appears convalescent.

She has had regular returns of the discharge until lately; it has varied both in quantity and regularity, and now seems as if it were about to cease; her health has been tolerable, except occasionally, when walking quickly, or otherwise exerting herself, she feels a little difficulty of breathing; she is under the necessity of taking some opening medicine occasionally, that the discharges from the system may be freely promoted.

I am, Gentlemen,
Your most obedient humble Servant,
JOHN KENWORTHY.

*Dobcross, Yorkshire,*

*Oct.* 16, 1810.

